# MIR-203A-3P AND MMP-2 PROTEINS ARE HIGHLY EXPRESSED IN CIRCULATING TUMOR CELLS FROM PATIENTS WITH PANCREATIC CARCINOMA

**DOI:** 10.1590/0102-672020210002e1628

**Published:** 2022-01-31

**Authors:** José Gabriel Rodríguez TARAZONA, Emne Ali ABDALLAH, Bianca de Cássia Troncarelli FLORES, Alexcia Camila BRAUN, Cláudia Malheiros Coutinho CAMILLO, Fabio Albuquerque MARCHI, Anna Paula Carreta RUANO, Ludmilla Thome Domingos CHINEN

**Affiliations:** 1 Hospital A.C. Camargo Cancer Center, Centro de Pesquisa Internacional - São Paulo - SP - Brasil

**Keywords:** Matrix Metalloproteinase 2, Neoplastic Cells, Circulating, Pancreatic Neoplasms, Thrombosis, Metaloproteinase 2 da Matriz, Células Neoplásicas Circulantes, Neoplasias Pancreáticas, Trombose

## Abstract

Ductal adenocarcinoma of the pancreas is the fourth most common cancer-associated cause of death in the Western world. The presence of circulating tumor cells (CTCs) can be considered a potential prognostic factor, as these cells represent tumor progression, allowing monitoring of therapeutic efficacy.

**OBJECTIVES:**

The objectives of this study were to explore the morphological, molecular, and phenotypic characteristics of CTCs from the blood of patients with pancreatic carcinoma and to correlate the findings with response to treatment, progression-free survival, overall survival (OS), and deep vein thrombosis (DVT).

**METHODS:**

Peripheral blood (10 mL) was analyzed before the beginning of treatment after 60 and 120 days. CTCs were detected by using ISET^®^ and characterized by immunocytochemistry. For microRNAs (miRNAs) analysis, peripheral leukocytes from the same patients and healthy individuals (controls) were collected in parallel at baseline. The expression of miRNAs was evaluated (in pool) using TaqMan® Array Human MicroRNA Cards v2.0.

**RESULTS:**

Only nine patients were included. The proteins, namely, matrix metalloproteinase-2 (MMP2) and TGFβ-RI, were highly expressed (77.7%) in CTCs at baseline; at the first follow-up, MMP2 was predominant (80%) and, at the second follow-up, MMP2 and vimentin were predominant (50%). Circulating tumor microemboli (CTMs) were found in two patients and both presented DVT. The miR-203a-3p was highly expressed in CTCs. The miR-203a-3p is involved in the stimulation of epithelial-to-mesenchymal transition (EMT) and is related to worse OS in pancreatic cancer (TCGA data).

**CONCLUSION:**

Due to the low number of patients and short follow-up, we did not observe a correlation between CTCs and response to treatment. However, there was a correlation between CTM and DVT and also miR-203a-3p was highly expressed in CTCs, corroborating the findings of EMT proteins. This study opens the perspectives concerning the dynamic change in the pattern of proteins expressed along with treatment and the use of miRNAs as new targets in pancreatic carcinoma.

## RESUMO -RACIONAL

O adenocarcinoma ductal do pâncreas é a quarta causa de morte associada ao câncer mais comum no mundo ocidental. A presença de células tumorais circulantes (CTCs) pode ser considerada um potencial fator prognóstico, visto que essas células representam a progressão tumoral, permitindo o monitoramento da eficácia terapêutica.

**OBJETIVOS:**

explorar as características morfológicas, moleculares e fenotípicas das células tumorais circulantes (CTCs) do sangue de pacientes com carcinoma pancreático e correlacionar os achados com a resposta ao tratamento, sobrevida livre de progressão, sobrevida global (SG) e trombose venosa profunda (TVP).

**MÉTODOS:**

o sangue periférico (10mL) foi analisado antes do início do tratamento e após 60 e 120 dias. As CTCs foram detectadas pelo ISET^®^ e caracterizadas por imunocitoquímica. Para análise de miRNAs, leucócitos periféricos dos mesmos pacientes e indivíduos saudáveis foram coletados em paralelo no início do estudo. A expressão de miRNAs foi avaliada usando *TaqMan T Array Human MicroRNA Cards* v2.0.

**RESULTADOS:**

foram incluídos 9 pacientes. As proteínas MMP2 e TGFß-RI foram altamente expressas (77,7%) nas CTCs no início do estudo. No primeiro acompanhamento, MMP2 era predominante (80%) e no segundo acompanhamento, MMP2 e vimentina eram predominantes (50%). Microêmbolos tumorais circulantes (MTC) foram encontrados em dois pacientes e ambos apresentavam TVP. O miR-203a-3p foi altamente expresso em CTCs. miR-203a-3p está envolvido na estimulação da transição epitelio-mesenquima (TEM) e relacionado a pior SG no câncer pancreático (dados TCGA).

**CONCLUSÃO:**

Devido ao baixo número de pacientes e curto seguimento, não observamos correlação entre CTCs e resposta ao tratamento. No entanto, houve uma correlação entre MTC e TVP. Além disso, miR-203a-3p foi altamente expresso em CTCs, corroborando os achados de proteínas EMT. Este estudo abre perspectivas sobre a mudança dinâmica no padrão de proteínas expressas ao longo do tratamento e a utilização de miRNAs como novos alvos no carcinoma pancreático.

## INTRODUCTION

Pancreatic cancer is currently ranked as the 14th most common cancer and the 7th leading cause of cancer mortality in the world. There is a general trend of higher incidence rates in developed countries^15^. Although not among the top 10 cancers in Brazil, it is the eighth leading cause of death from cancer, due to diagnosis in locally advanced or metastatic stage of the disease^4^ ^,^ ^20^. The most common and also most aggressive histological subtype of pancreatic cancer is the pancreatic ductal adenocarcinoma (PDAC), which represents 85-90% of all pancreatic neoplasms^3^. Surgical resection continues as the only treatment modality with a potential for the cure; however, only 15-20% of patients with pancreatic cancer diagnosed with the disease are operable. However, the level of recurrences after surgery is still high, both local and systemic. Adjuvant treatment is used to improve survival prospects and involves chemotherapy, radiation, and/or combined modalities, but there are still controversies regarding the treatment of choice^1^.

CA19-9 has been applied as the “gold standard” for monitoring and diagnosing patients with pancreatic cancer^10^, due to its low positive predictive value, making it suitable only for monitoring response to treatment and as a marker of disease recurrence. The lack of a validated and specific biomarker for this disease remains a major challenge^23^.

Circulating tumor cells (CTCs) leak from tumor into the circulation and can potentially invade distant tissues to form evident metastases. While most CTCs are single cells, a small fraction travels as group of cells^2^. Circulating tumor microemboli (CTMs) are defined as a cluster or group of CTCs containing three or more distinct nuclei^11^. CTM appears to have greater metastatic potential than individual CTC in circulation^14^.

A crucial step toward intravasation and survival in the bloodstream is to obtain plasticity and motility, which involves the epithelial-to-mesenchymal transition (EMT) process and both CTCs and CTMs can go through this process ^9^, which can be regulated by microRNAs (miRNAs).

miRNAs form complex networks that regulate cell differentiation, development, and homeostasis. The deregulation of its function is associated with an increasing number of human diseases, mainly cancer, being, therefore, prognostic markers or potential targets for new therapies against cancer^1^. Some miRNAs interact with the set of critical molecules involved in EMT engineering, modulating its expression and, consequently, its function^12^.

Thus, this study aimed to evaluate the expression of proteins related to EMT and the expression of miRNAs in CTCs from patients with pancreatic carcinoma and to correlate these findings with clinical data, deep vein thrombosis (DVT), progression-free survival (PFS), and overall survival (OS).

## METHODS

### Ethical statement

*The protocol was approved by the* Research Ethics Committee of the A.C. Camargo Cancer Center (code 2388/17) *in accordance with the ICH-GCP guidance.*

### Study design

This was a prospective longitudinal single-center study performed by collecting whole blood from patients with pancreatic carcinoma and undergoing treatment with chemotherapy, immunotherapy, or target therapy.

Blood samples (10 mL) were collected before the beginning of treatment, after 60 and 120 days, with imaging tests. As a negative control, blood from healthy individuals was used.

This project was submitted to the Research Ethics Committee of the A.C. Camargo Cancer Center (code 2388/17). The samples were collected by accepting and signing the informed consent form.

The inclusion criteria were as follows: patients with histological diagnosis of locally advanced or metastatic pancreatic carcinoma, patients above 18 years; patients who underwent the first line of treatment, metastatic disease confirmed by pathological and/or radiological evaluation, extent of the disease determined by clinical examination and imaging, and disease measurable by the RECIST version 1.1 criteria (*Response Evaluation Criteria in Solid Tumors*).

Patients who underwent cancer treatment earlier were excluded.

### Isolation of CTCs and analysis of markers

To separate the CTCs, the method of filtration by size through the ISET^®^ device (Isolation by SizE of Tumor cells; Rarecells, France) was used. The peripheral blood samples of the patient were collected in EDTA tubes and diluted to perform red blood cell lysis in 1:10 with *ISET Buffer* ^*TM*^ . After 10 min of homogenization, the samples were deposited in *ISET Block* ^*TM*^ , which contains a polycarbonate membrane with circular pores of 8 μm in diameter. The equipment filters the samples by vacuum aspiration and then the membranes are washed with phosphate-buffered saline (PBS) 1× (pH: 7.3), removed from the *ISET Block* ^*TM*^ , and, when dried, stored at −20°C. Of the 10 mL of blood analyzed, 6 mL were destined for cytopathological analysis and 4 mL for molecular analysis (submerging the membrane in later RNA and subsequent DNA and RNA extraction).

To characterize CTCs that were expressing the proteins evaluated in this study, we performed an immunocytochemistry (ICC) assay. Important proteins in the EMT were searched: anti-TGFβ-R1 (Cusabio 1:100, code: CSB-PA061850), anti-Mesothelin (Sigma-Aldrich 1:50, code: SAB5500143), anti-Vimentin (Cusabio 1:100, code: CSB-PA025857LA01HU), and anti-matrix metalloproteinase-2 (MMP2) (Cusabio 1:100, code: CSB-PA06879A0Rb). For the ICC reaction positive controls, we used cell lines spiked in health blood samples and filtered through ISET^®^. For anti-TGFβ-R1 antibody control, we used the cell line A-549; for anti-Mesothelin antibody we used the Hela cell line; for anti-Vimentin we used the MCF7 cell line; and for anti-MMP2, we used the U87 cell line, according to The Human Protein Atlas (http://www.proteinatlas.org/). As a negative control of ICC, we used the same cell line, omitting the primary antibody, to ensure that cross-reactivity was excluded. To confirm that the CTCs analyzed were not leukocytes, we used the anti-CD45 antibody (Sigma-Aldrich 1:100, code: HPA000440).

The ICC was performed with double marking, using the GBI Labs Golden Bridge International Kit (GBI Labs.) with the desired markers. The ISET membrane spots were cut and placed in 24-well plates. Antigenic recovery was performed by adding 1 mL of 1× target retrieval solution (Dako™) from each well by heating in a microwave container with distilled water for approximately 6 min with cooling intervals of every 1 min and 40 s. Each spot was hydrated with 160 μl of 1× Tris-buffered saline (TBS; pH: 7.3) for 20 min. The cells were permeabilized with 160 μL of 0.2% TBS + Triton X-100 for 5 min at room temperature. After a new wash with TBS, the membranes were incubated for 15 min in the dark and at room temperature, with hydrogen peroxide, and washed again with TBS. Then, the primary antibody was applied to the spots and incubated overnight. The membranes were washed again with TBS, incubated for 30 min in polymer/HRP (Dako, Santa Clara, CA, USA), washed again, and revealed by the DAB chromogen of the same kit. Then, membranes were washed with TBS, followed by 2-h incubation with the second primary antibody and a 30-min incubation with the AP polymer (GBI Labs). The second antibody was revealed by Permanent Red kit (GBI Labs) and previously diluted according to the instructions of the manufacturer for 10 min. Finally, the spot was washed with distilled water and stained with hematoxylin for 2 min, washed again with distilled water, and adhered to the slides for reading under a light microscope using PBS.

The slides were examined under a white light microscope, Olympus BX61 (Tokyo, Japan), coupled to a high-resolution digital camera SC100-Olympus (Tokyo, Japan). CTCs were characterized according to the following criteria: nuclear size=16 μm, irregularity of the nuclear contour, presence of visible cytoplasm, and high nucleus-to-cytoplasm ratio (>0.8). When any of the described criteria were missing, the cells were classified as atypical. The results were given in the number of CTCs per milliliter of blood, according to statistical analysis performed by Krebs et al., counting CTCs in four membrane spots or more (12). After cytopathological analysis, the CTCs values were added and the mean was calculated. Thus, we had the CTCs calculations in 1 mL of blood. In addition, the presence and absence of CTMs were included in the analysis.

### miRNA expression analysis

#### RNA isolation

RNA extraction was performed using the AllPrep DNA/RNA/miRNA Universal Kit (Qiagen, Hilden, Germany). Briefly, four membrane spots without formaldehyde were cut into small fragments (~2 mm^2^) in 1.5-mL microtubes, and the cells were lysed using the RLT Plus buffer (Qiagen) and mixed for 1 min. Subsequently, the steps were followed according to the instructions of the manufacturer.

The RNA concentration was measured on the Nanodrop™ ND-1000 spectrophotometer (Thermo Scientific) using 1.5 μl of sample and checking the absorbance at 260 nm.

### Real-time RT-PCR (qPCR)

To verify the quality of the extracted RNA, a small nuclear RNA U6B (RNU6B) was amplified in all samples. The specific cDNA synthesis reaction was performed according to the procedures recommended in the TaqMan^®^ MicroRNA Assays manual (Applied Biosystems). Only samples that presented a consistent amplification curve were used to analyze the miRNA profile.

The determination of miRNA expression profiles was performed using the TaqMan™ Array Human MicroRNA A Cards v2.0 methodology (containing 372 miRNAs and 7 controls) according to the *Megaplex™ Pools for MicroRNA Expression Analysis kit* (Applied Biosystems). The synthesis and preamplification of the cDNA were performed using 150 ng of RNA from leukocytes from volunteers without neoplasia (pool of five samples), RNA from leukocytes from patients with pancreatic cancer (pool of six samples), and RNA from CTCs of patients with pancreatic cancer (pool of nine samples). The amplification of the miRNAs was performed using the 7900HT Fast Real-Time PCR System (Applied Biosystems).

The results were analyzed using the software RQ Manager version 1.2 (Applied Biosystems). The level of expression of the miRNAs was quantified in relation to the expression of a reference miRNA and was normalized according to the calibrator sample (leukocyte sample pool from healthy volunteers). The miRNA used as a reference was miR-126, selected through the NormFinder tool (https://moma.dk/normfinder-software), assessing which miRNA has the most stable expression in a set of samples. The relative quantification (Rq) was calculated using the ΔΔCT method^13^.

The miRNAs were selected according to the greatest difference in expression between the CTC and leukocyte groups, assessed by fold-change=2 for increased expression and fold-change=−2 for decreased expression. For the construction of the boxplot, the ggplot2 package available for the R program was used.

### Statistical analysis

A descriptive analysis of each group (those that express EMT-related proteins versus those that do not) was performed in relation to the clinical-pathological variables. To assess differences and associations between groups, the chi-square method was used for categorical variables. To analyze PFS and OS, the Kaplan-Meier method was used and the difference between the curves was calculated by log rank. All statistical analysis was performed using the SPSS program for Windows, version 15. The p-value <0.05 was considered significant.

For the calculation of OS, we considered the time between the first sampling of the patient until his death and for PFS, we considered the time between the first sampling of the patient and the objective progression of tumor. The baseline (first sample) of survival analyzes in this study was carried out from the date of the first collection of CTCs.

## RESULTS

### Clinical-pathological characteristics

We collected samples from 10 patients with pancreatic adenocarcinoma before the surgical procedure (baseline) between May 2018 and September 2019. One patient was excluded from the study because pathological analysis confirmed that it was a neuroendocrine tumor. The first follow-up was carried out 2 months after the baseline collection in five patients, and we lost four patients (death of one patient and loss of follow-up of three patients). The second follow-up (4 months after baseline collection) was performed in eight patients.

Of the nine patients included, 4 (44.44%) were men and 5 (55.55%) were women; the median age was 59.7 years (42-82). Healthy donors (n=7), six women and one man, with a median age of 48 years, were also recruited as a control group for the analysis of the miRNA expression experiments.

Regarding the histological grade, out of nine, 6 (66.66%) had ductal adenocarcinoma, 2 (22.22%) had adenocarcinoma, and in 1 (11.11%), tumor was classified as carcinoma.

Of the nine patients included, three (33.33%) progressed and six patients remained at the same stage of the disease at the time of the last follow-up. Of the three patients who progressed, two patients had nonmetastatic disease and one patient has metastatic disease.

**Figure 1 -:**
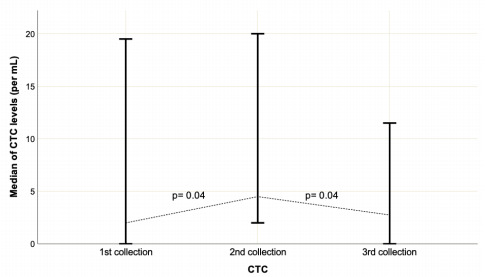
Median of CTCs/mL in the three collections. From the Wilcoxon test, we observed that the CTCs in the whole group increased with a statistically significant difference between the first and the second collections (p=0.04) and between the second and third collections, and they decreased with a statistically significant difference (p=0.04).

Patients who had experienced progression had a greater distribution of CTCs at the baseline (p=0.54). The differences in the levels of CTCs throughout the treatment are shown in Figure 1.

Regarding the pathological staging of the patients included in this study, 4 (44.44%) started the study in stage IV, 2 (22.22%) in stage III, 2 (22.22%) in stage IB, and 1 (11.11%) in stage IIB. Regarding metastases (pM), 5 (55.55%) patients were M0 and 4 (44.44%) were M1.

Concerning the therapeutic strategy established for the patients in this study, it was found in the medical records that 4 (44.44%) patients started palliative treatment, 3 (33.33%) patients started neoadjuvant treatment, and 2 (22.22%) patients started curative treatment. The other clinical and pathological characteristics of the patients are given in Table 1.

**Table 1 -:** Clinical and pathological characteristics of the study patients (n=9). TNM staging according to AJCC.

Variables (n)	Nº	%
Total number of patients	9	100
Age at study entry, in years (9)		
Average	57	
Median	59.7	
Variation	42-82	
Gender (9)		
Male	4	44.44
Female	5	55.55
Histological grade (9)		
Ductal adenocarcinoma	6	66.66
Adenocarcinoma	2	22.22
Carcinoma	1	11.11
Primary site diagnosis (9)		
Malignant neoplasm of pancreas	9	100
Metastases in diagnosis (9)		
Yes	4	44.44
No	5	55.55
Progression (9)		
Yes	3	33.33
No	6	66.66
Thrombosis (9)		
Deep venous thrombosis	2	22.22
Data not available	2	22.22
Without deep venous Thrombosis	5	55.55
Tumor size (pT) (9) baseline		
T2	2	22.22
T3	1	11.11
T4	6	66.66
Lymph node involvement (pN) (9) baseline		
N0	4	44.44
N1	5	55.55
Metastases (pM) (9) baseline		
M0	5	55.55
M1	4	44.44
Pathological staging (9)		
IB	2	22.22
III	2	22.22
IIB	1	11.11
IV	4	44.44
Therapeutic strategy (9)		
Neoadjuvant	3	33.33
Palliative	4	44.44
Curative	2	22.22

Within the biomarkers used in this study, by medical record data, we observed that the median level of CA 19-9 in the baseline collection (first collection) was 337.9 IU/mL (3.1-41544 IU/mL).

### Protein expression in CTCs

We evaluated the expression of proteins involved in the EMT process by ICC in the CTCs of nine patients in the first collection, five patients in the second collection, and eight patients in the third collection.

We analyzed the protein expression of CTCs separately, relating each marker to each collection and what we could observe was the expression of MMP2 in 77.77% of the cases of the first collection (C1). In fact, patients with MMP2 expression in CTCs at C1 had a higher distribution of CTC levels but without statistical significance (p=0.33; Figure 1).

**Figure 2 -:**
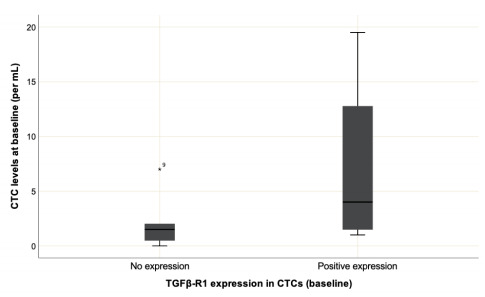
Distribution of CTCs in protein expression of TGFβ-R1 in the first collection. It has no statistical significance (p=.09). The quantitative distribution of variables was calculated using the Mann-Whitney U test.

TGFβ-R1 was found to be expressed in 44.44% of cases. Patients with TGFβ-R1 expression in CTCs at C1 showed a higher distribution of CTCs levels, without statistical significance (Figure 2; p=0.09) also known as Vimentin.

There was no statistically significant difference in the number of cases with the presence of proteins or correlation with the presence of distant metastasis, or with disease progression at any time analyzed. Protein expressions in CTCs are given in Table 2 and the relationship of the markers with clinical evolution is given in Table 3.

**Table 2 -:** Data of the study biomarkers, CTC detection rate, and markers in CTCs at all times of the study (n=9).

Variables (n)	Nº	%
Biomarkers		
Median level of CA 19-9 (IU/mL) baseline	337.9 (3.1-41544)	
Average level of CA 19-9 (IU/mL) baseline	7841.7 (3.1-41544)	
Median of CTCs/mL		
First collection (evaluated on 9/9)	2 (0-19.5)	
Second collection (evaluated on 5/9)	4.5 (2-20)	
Third collection (evaluated on 9/9)	2.75 (0-11.5)	
Patients with the presence of CTCs		
First collection (evaluated on 9/9)	8	88.88
Second collection (evaluated on 5/9)	5	55.55
Third collection (evaluated on 9/9)	7	77.77
Total collections (22)	20	90.90
Patients with MMP2-positive circulating tumor cells		
First collection (evaluated 9)	7	77.77
Second collection (evaluated 5)	4	80
Third collection (evaluated 8)	4	50
Patients with vimentin-positive circulating tumor cells		
First collection (evaluated 9)	3	33.33
Second collection (evaluated in 5)	3	60
Third collection (evaluated in 8)	2	25
Patients with TGFβ-R1-positive circulating tumor cells		
First collection (evaluated 9)	4	44.44
Second collection (evaluated 5)	3	60
Third collection (evaluated 8)	1	12.5
Patients with circulating tumor microemboli		
First collection (evaluated 9)	1	11.11
Second collection (evaluated 5)	0	0
Third collection (evaluated 8)	1	12.5

CA 19-9, carbohydrate antigen 19-9.

**Table 3 -:** Clinical and protein expression characteristics of the patients involved in this study.

Pte	Gn	Phase	T	N	M	Histological type	M Dx	M site	1st coll. CTC/Ml	Protein expression	2nd coll. CTC/mL	Protein expression	3rd coll. CTC/mL	Protein expression	CTM	CA 19-9 to Dx	Prog	Treatment	Thrombosis	Chemotherapy
1	F	III	4	0	0	Carcinoma	No		2	VIM, TGFβ	7	MESOTH, VIM, TGFβ	3.5	MMP2, VIM, TGFβ	Yes	211	No	Neoadjuvant	DVT MSD	mFOLFIRINOX
2	M	III	4	0	0	Ductal adenocarcinoma	No		2	MMP2	4.5	MMP2, VIM, TGFβ	1	MMP2	No	337.9	No	Neoadjuvant	No data	FOLFIRINOX
3	F	IB	2	0	0	Ductal adenocarcinoma	No		1	MMP2, TGFβ	2	MMP2	0	-	No	26.5	Yes	Neoadjuvant	No data	mFOLFIRINOX
6	F	IB	2	0	0	Ductal adenocarcinoma	No		19.5	MMP2, MESOTH, VIM, TGFβ	Collection loss	-	2.5	Negative CTCs	No	39	No	Curative	Without DVT	FOLFOX
10	M	IIB	3	1	0	Ductal adenocarcinoma	No		7	MMP2	Patient death	-	Patient Death	-	No	388.5	Yes	Curative	Without DVT	DOES NOT APPLY
5	M	IV	4	1	1	Adenocarcinoma	Yes	Liver	6	MMP2, VIM, TGFβ-RI	Collection loss	-	3	Negative CTCs	No	3,1	Yes	Palliative	Without DVT	mFOLFIRINOX
7	M	IV	4	1	1	Adenocarcinoma	Yes	Liver, Peritoneum	1.5	MMP2, MESOTH	Collection loss	-	1	MMP2	No	41544	No	Palliative	Without DVT	mFOLFIRINOX
8	F	IV	4	1	1	Ductal adenocarcinoma	Yes	Liver	0.5	MMP2	4	MMP2	3	VIM	Yes	6700	No	Palliative	DVT LLL	mFOLFIRINOX
9	F	IV	4	1	1	Ductal adenocarcinoma	Yes	Liver, Lymph Nodes	0	-	20	MMP2, VIM, TGFβ	11.5	MMP2	No	21325.9	No	Palliative	Without DVT	mFOLFIRINOX

Pte: patient; Gn: genre; F: female; M: male; T: tumor; N: lymph nodes; M: metastasis; Dx: diagnosis; VIM: vimentina; TGFβ: transforming growth factor beta receptor 1; MMP2: matrix metalloproteinase-2; MESOTH: mesothelin; Coll: collection; CTMs: circulating tumor microemboli; CA 19-9: carbohydrate antigen 19-9; Prog: progression; DVT: deep vein thrombosis; URM: upper right member; LLL: left lower limb. This table explains the conditions of nonmetastatic and metastatic patients. We found that patients with CTMs had DVTs.

### Expression of miRNAs

Table 4 shows the 14 best miRNAs selected in this study, 13 with less expression in CTCs compared with the same patient leukocytes and 1 with higher expression in CTCs after the same comparison in patients with leukocytes.

**Table 4 -:** Comparison between miRNAs differentially expressed in CTCs and in primary tumors obtained in TCGA.

miRNAs (CTCs)	FC	p-value	Study	Status
miR-132-5p*	−1.72	1.78E-02	−38.64	Low
miR-143-5p*	−1.80	4.44E-02	−26.28	Low
miR-197-3p*	−1.88	7.41E-04	−21.87	Low
miR-142-3p*	−5.26	5.08E-05	−8.66	Low
miR-223-3p*	−3.93	2.90E-03	−7.29	Low
miR-223-5p*	−2.70	3.06E-03	−7.29	Low
miR-142-5p*	−3.48	1.50E-03	−6.93	Low
miR-145-5p*	−2.23	3.56E-03	−6.67	Low
miR-145-3p*	−1.75	1.81E-02	−6.67	Low
miR-22-5p*	−2.94	9.06E-06	−6.06	Low
miR-345-5p*	−2.11	2.62E-03	−4.03	Low
miR-340-3p*	−2.22	2.18E-04	−3.94	Low
miR-340-5p*	−1.74	3.46E-02	−3.94	Low
miR-150-5p	−3.73	2.26E-02	−2.86	Low
miR-150-3p	−2.56	2.19E-03	−2.86	Low
miR-191-5p	−2.46	1.28E-05	−2.72	Low
miR-342-3p	−2.80	3.64E-04	−2.66	Low
miR-18a-5p	−2.07	3.22E-03	−2.63	Low
miR-18a-3p	−1.46	1.47E-02	−2.63	Low
miR-590-5p	−1.88	3.81E-03	−2.54	Low
miR-362-3p	−1.83	4.77E-03	−2.26	Low
miR-324-5p	−1.92	9.09E-03	−2.11	Low
miR-28-3p	−1.59	4.86E-02	−2.05	Low
miR-28-5p	−1.43	2.62E-02	−2.05	Low
miR-203a-3p*	3.90	3.53E-02	35.80	High

T: tumor; NT: normal tissue; FC: fold-change. The asterisk (*) indicates the miRNAs that showed the expression profile with the same direction of the miRNAs identified in this study. Fourteen miRNAs agreed with the expression between CTCs and tumor (13 downexpressed and 1 overexpressed).

**Figure 3 -:**
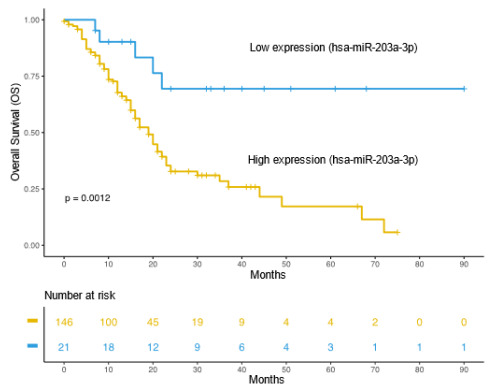
The increased expression of miR-203a-3p is associated with poor overall survival in patients with pancreatic cancer with initial disease stage after analysis with TCGA data.

The obtained samples were 182 pancreatic cancer samples from TCGA (The Cancer Genome Atlas) and selected for the evaluation of miRNAs differentially expressed between neoplastic and non-neoplastic samples. Only samples from patients with the early pathological stages (I and II) were considered, resulting in 50 samples. We considered significant miRNAs with p-value <0.05 and fold-change=2 and =−2. The 93 selected targets were compared with those obtained in this study, resulting in 25 candidates. It was observed that miR-203a-3p was associated with poor OS in patients with early diagnosis of the disease. It can be verified that the increased expression of the miR-203a-3p is related to poor OS in patients with early-stage pancreatic cancer (Figure 3). This analysis was conducted with the survminer R package (https://github.com/kassambara/survminer/) from 167 early-stage pancreatic cancer samples that presented available information for the survival curve.

## DISCUSSION

This study found CTCs in all nine patients included with pancreatic cancer. MMP-2 was found to be highly expressed in these cells, and also there was a correlation between the presence of CTM and DVT, which opens perspectives in the monitoring of patients with pancreatic cancer, so prone to DVT. As we expected, we found an EMT-related miRNA highly expressed in these CTCs of patients, that is, miR-203a-3p. Therefore, despite the little genetic material, due to the low number of CTCs isolated from patients, we obtained important information, which can serve as new therapeutic targets. We believe that the model of the sample pool of our experiment allows to dimension targets that in only patient could not be found, due to the diversity and heterogeneity of the CTCs.

MMPs are a family of proteolytic enzymes that degrade multiple components of the extracellular matrix, including those in the basement membrane of the vessels. They are involved in tumor invasion, neoangiogenesis, and metastasis^22^. MMP2 (gelatinase A) breaks down type IV collagen, gelatin, elastin, and proteoglycans. It is regulated in many types of cancer, such as glioblastomas, melanomas, breast cancer, and colon cancer, promoting invasion and metastasis. Inhibition of MMP2 has been shown to cause radiosensitization, a decrease in tumor growth, and invasiveness, identifying this enzyme as an interesting target for the development of diagnostic and therapeutic approaches^17^.

Murray et al. ^16^ studied the expression of MMP2 in circulating prostate cells (CPCs), disseminated tumor cells (DTCs), and micrometastases (mMs) in the bone marrow of men with prostate cancer by ICC. In 185 patients with nonmetastatic cancer, CPCs were detected in 62.7%, DTCs in 62.2%, and mMs in 71.4%. In 30 patients with metastatic cancer, 100% of CPCs, DTCs, and mMs were detected. In all CPCs, DTCs, and mMs, the expression of MMP2 was positively associated with an increase in the Gleason score. The expression of MMP2 in CPCs and DTCs showed agreement. Our findings, although in pancreatic cancer, demonstrated that MMP2 expression was the highest of all study proteins in all collections, being the highest expression found in the second collection, in 80% of patients.

Poruk et al. ^18^ studied the peripheral and portal blood samples obtained from 50 patients with PDAC before surgical resection and filtered using ISET. CTCs were identified by immunofluorescence for cytokeratin and vimentin positivity. Cytokeratin was found to be expressed in 78% of patients and vimentin in 67%. The detection of CTCs expressing both vimentin and cytokeratin was predictive of recurrence (p=0.01). In this study, vimentin was not highly expressed in the first collection, although its expression in the second collection increased, which may lead to the hypothesis that it is a mechanism of resistance to chemotherapy after the start of chemotherapy.

In a previous study by our group, Gasparini-Junior et al. ^8^ studied TGFβ-RI expression in patients with pancreatic ductal adenocarcinoma and 16 patients were tested for TGFβ-RI, 4 showing positive marking (25% of patients). There was no statistically significant difference in SLP between patients who showed positive marking for TGFβ-RI compared with those who did not. However, when TGFβ-RI and MMP-2 were evaluated together, those patients who presented either or both (mesenchymal markers) on CTCs progressed more quickly than those without any mesenchymal markers (2.84 vs. 5.43 months), although with no statistically significant difference (p=0.14). This study allowed us to observe the kinetics of this protein in the three collections, noticing an increase in the second moment, presenting in 60% of the patients, and with an important drop in expression in the third moment of the collections, which may expose that the mesenchymal phenotype is more exhibited after the beginning of chemotherapy treatment, it can, maybe, represent a tumor resistance mechanism.

Vimentin, a major constituent of the intermediate filament protein family, is ubiquitous in normal mesenchymal cells and is known to maintain cell integrity and provide resistance against stress. Increased vimentin expression has been reported in various epithelial cancers (prostate, breast, melanoma, lung, gastrointestinal, and CNS tumors), correlating with increased tumor growth, invasion, and poor prognosis. However, the role of vimentin in cancer progression remains unclear, despite being widely used as a marker for EMT ^19^. Higher expression of vimentin in pancreatic cancer cells may imply a higher state of malignancy^24^.

A recent study analyzed whether vimentin on the cell surface could be a biomarker to isolate CTCs in PDAC and observed that this protein was highly expressed in pancreatic tumor cells with a mesenchymal phenotype. Positive CTCs for vimentin (CTCvim) were detected in 76 of 100 patients with PDAC, using a microfluidic assay. CTCvim counts were correlated with changes in tumor burden for patients undergoing resection. Significantly reduced CTC counts were seen after chemotherapy in patients who responded to treatment. Higher CTC counts in the preoperative period were correlated with lower recurrence-free survival. Together, vimentin and CTCs can be used as trusted biomarkers in PDAC^21^.

Corroborating our findings with miR-203a-3p, ^6^ performed small RNA sequences of an epithelial breast cell line (D492) and its mesenchymal derivative (D492M) grown in a three-dimensional microenvironment. Among the most regulated miRNAs in D492M was miR-203a, a miRNA that plays an important role in epithelial differentiation. The increased expression of miR-203a was seen in D492. When miR-203a was overexpressed in D492M, a partial reversal toward the epithelial phenotype was observed. The analysis of gene expression of D492M and D492MmiR-203a revealed peroxidasin, which was involved in the production of collagen IV, as the most significantly regulated gene in D492MmiR-203a. Overexpression of miR-203a in D492M induced partial TME and reduced peroxidasin expression. In addition, the authors demonstrated that miR-203a is a new peroxidasin repressor. The MiR-203-peroxidasin axis can be an important regulator of EMT/MET and remodeling of the basement membrane. In CTCs, this miRNA may be involved in these mechanisms, and additional studies are needed to better understand this pathway and its role in CTCs from patients with pancreatic cancer.

Chen et al. ^7^ aimed to provide a new therapeutic target (LINC00342) for NSCLC therapy. Target expression was detected by real-time PCR (qRT-PCR) Cell migration and invasion were measured by Transwell assay. The DIANA tools of the online software were used to predict the connection of the LINC00342 and miR-203a-3p by luciferase. LINC00342 expression was increased in tissues and NSCLC cells compared with normal tissues and cells. The elimination of LINC00342 suppressed cell proliferation, colony formation, migration, and invasion. The study suggested that LINC00342 contributes to the growth and metastasis of NSCLC cells through the competitive targeting of miR-203a-3p.

## CONCLUSION

Despite the super-restricted cohort, our data open the perspectives for new ways of evaluating CTCs, not only analyzing them under the cytopathological aspect, but also at the miRNA level. This associated information, in a well-designed study, may be useful in the clinical and therapeutic management of patients with pancreatic adenocarcinoma.
